# The occurrence of immune-related adverse events is an independent risk factor both for serum HBsAg increase and HBV reactivation in HBsAg-positive cancer patients receiving PD-1 inhibitor combinational therapy

**DOI:** 10.3389/fimmu.2024.1330644

**Published:** 2024-03-15

**Authors:** Yingfu Zeng, Jiwei Huang, Jiahui Pang, Shufang Pan, Yuankai Wu, Yusheng Jie, Xinhua Li, Yutian Chong

**Affiliations:** ^1^ Department of Infectious Diseases, The Third Affiliated Hospital of Sun Yat-sen University, Guangzhou, China; ^2^ Guangdong Provincial Key Laboratory of Liver Disease Research, The Third Affiliated Hospital of Sun Yat-sen University, Guangzhou, China; ^3^ Department of Pharmacy, The Third Affiliated Hospital of Sun Yat-sen University, Guangzhou, China

**Keywords:** cancer, PD-1 inhibitor, HBsAg loss, HBsAg increase, HBV reactivation, immune-related adverse events, risk factor identification

## Abstract

**Background:**

Previous studies have suggested the potential of PD-1/PD-L1 inhibitors in the treatment of chronic HBV infection. However, since phase III clinical trials have not yet been announced, additional clinical insights may be obtained by observing changes in serum hepatitis B surface antigen (HBsAg) and HBV-DNA levels in cancer patients undergoing PD-1 inhibitor therapy.

**Objective:**

To explore the effects of PD-1 inhibitor combinational therapy on serum HBsAg and HBV-DNA levels, investigate the incidence of HBsAg loss, HBV reactivation (HBVr), and immune-related adverse events (irAEs), and identify the risk factors associated with significant HBsAg fluctuations and HBVr.

**Methods:**

A retrospective study including 1195 HBsAg-positive cancer patients who received PD-1 inhibitors between July 2019 and June 2023 was conducted, and 180 patients were enrolled in this study. Serum HBsAg levels before and after PD-1 inhibitor administration were compared across different subgroups. The Pearson χ^2^ or Fisher exact test was performed to investigate the relationships between categorical variables. Univariable and multivariable analysis were performed to identify the risk factors associated with significant HBsAg fluctuations and HBVr.

**Results:**

With the concurrent use of antiviral agents, serum HBsAg levels decreased (Z=-3.966, P < 0.0001) in 129 patients and increased (t=-2.047, P=0.043) in 51 patients. Additionally, 7 patients (3.89%) achieved serum HBsAg loss. Virus replication was suppressed in most of the enrolled patients. When divided patients into different subgroups, significant HBsAg decreases after PD-1 inhibitor administration were discovered in lower baseline HBsAg group (Z=-2.277, P=0.023), HBeAg-seronegative group (Z=-2.200, P=0.028), non-irAEs occurrence group (Z=-2.007, P=0.045) and liver cancer group (Z=-1.987, P=0.047). Of note, 11 patients and 36 patients experienced HBVr (6.11%) and irAEs (20%), respectively, which could lead to discontinuation or delayed use of PD-1 inhibitors. After multivariable analysis, HBeAg-seropositive (OR, 7.236 [95% CI, 1.757-29.793], P=0.01) and the occurrence of irAEs (OR, 4.077 [95% CI, 1.252-13.273], P=0.02) were identified as the independent risk factors for significant HBsAg increase, the occurrence of irAEs (OR, 5.560 [95% CI, 1.252-13.273], P=0.01) was identified as the only independent risk factor for HBVr.

**Conclusion:**

PD-1 inhibitors combined with nucleos(t)ide analogues (NAs) may exert therapeutic potential for chronic HBV infection in cancer patients. However, attention also should be paid to the risk of significant elevation in HBsAg levels, HBVr, and irAEs associated with PD-1 inhibitor combinational therapy.

## Introduction

Immune checkpoint inhibitors (ICIs) have shown dramatic improvement in clinical outcomes compared with standard therapy for a range of cancer types in recent years, it enhances antitumor immunity by targeting intrinsic down regulators of immunity, such as programmed cell death 1 (PD-1) or its ligand, programmed cell death ligand 1 (PD-L1) (1). Except for the critical roles of CD8^+^ T cells in anti-tumor immunity upon PD-1/PD-L1 blockades (2), CD4^+^ T cells are also demonstrated to be required for efficacious anti-tumor responses, such as the percentages of naive CD4^+^ T cells secreting certain cytokines including IFN-γ and TNF-α before receiving nivolumab, were significantly higher in patients with better response to anti-PD-1 therapy (3). Similar to cancer patients, T cells are also described as”exhausted” or functionally impaired and unable to proliferate or secrete antiviral cytokines (IFN-γ) in chronic hepatitis B (CHB) (4), and emerging evidences suggest that the same checkpoint pathways may play a crucial role during acute (5) and chronic (6) hepatitis B virus (HBV) infection.

Failure to eliminate covalently closed circular DNA (cccDNA), which is the nuclear reservoir of the virus, is a major barrier to the cure of chronic HBV infection. It seems plausible that the induction of functional HBV‐specific T cells is a good approach for HBV clearance since virus-specific T cells are capable of removing cccDNA‐carrying cells in about 90% of infected patients (7). Consistent with this concept, previous studies have shown that the blockade of PD-1/PD-L1 may improve HBV-specific T-cell function *in vitro* (8–10). Besides, a phase Ib study in 2019 has noticed that 20 of the 22 patients (90.91%) who received nivolumab have a reduction in serum hepatitis B surface antigen (HBsAg), and nivolumab is well-tolerated in hepatitis B e antigen (HBeAg)-seronegative CHB patients (11). And in 2022, a phase IIb clinical trial (NCT04465890) of ASC22 (Envafolimab), a PD-L1 inhibitor, in patients with CHB reported that 7 patients with baseline HBsAg ≤ 500 IU/ml experienced HBsAg reduction > 0.5 log10 IU/ml under ASC22 and NAs, 3 patients even had HBsAg seroclearance (undetectable, < 0.05 IU/ml). However, more immune-related adverse events (irAEs) occurred in the ASC22 group (12). Hitherto, the implementation of phase III clinical trials of PD-1 or PD-L1 inhibitors in the treatment of CHB is yet to be announced.

Despite the exhilarating and promising study results, previous studies also have shown that PD-1 inhibitor monotherapy or combined with other ICIs (immune checkpoint inhibitors) pose a risk of HBV reactivation (HBVr) (13, 14), lack of prophylaxis antiviral treatment (15, 16), undetectable HBV-DNA (16), and combined with hepatic artery intubation chemotherapy (HAIC) (17) were identified as independent risk factor for HBVr. In addition to the impressive anti-tumor effects of ICIs, a spectrum of unique side effects referred to as irAEs have been reported (18). The mechanism of this may be that ICIs enhance the activity of T cells against antigens expressed in tumors and healthy tissues, and increase pre-existing levels of autoantibodies and inflammatory factors (1). It’s indicated that an overall incidence of irAEs ranges between 27%-78% in phase III trials of anti-PD-1/PD-L1 agents in cancer patients (19, 20). Aside from the possible permanent effects on the endocrine system, most of the irAEs are reversible. Deaths from irAEs are rare, however, deaths due to myocarditis, pneumonitis, colitis, and neurologic events, among others, can occur (1).

To improve objective responsive rate (ORR), ICI monotherapy was less received by cancer patients, and combination therapies including different types of ICIs, targeted agents, chemotherapy, and interventional therapies (21–24) were commonly used. However, the incidence of HBVr in cancer patients with ongoing PD-1 inhibitor combination therapies remains unclear, and more research is needed to validate the relationship between PD-1 inhibitors and immune-mediated clearance of HBV or serum HBsAg clearance in this context. Besides, whether there is a certain correlation between the occurrence of irAEs and changes in HBV serologic markers also needs to be clarified. In our study, each enrolled patient needed to be carefully investigated by two clinicians whether they had experienced irAEs before the first or second study endpoint, which were described in the study design, and concurrent use of NAs was required. This study aims to observe the changes in serum HBsAg and HBV-DNA levels in HBsAg-positive cancer patients, particularly significant increases or decreases in HBsAg levels. Meanwhile, investigating the incidence of HBsAg loss, irAEs, HBVr, and identifying the risk factors associated with HBsAg fluctuations and HBVr in cancer patients.

## Materials and methods

### Patients

This retrospective study was conducted with the approval of the institutional review board and was conducted following the Declaration of Helsinki. The requirement for written informed consent was waived because of the retrospective nature of this study. 1195 HBsAg-positive Cancer patients who were treated with PD-1 combinational therapy between July 2019 and June 2023 were identified. Data were collected through a manual review of patient electronic medical records, and laboratory and imaging results database by 2 reviewers. Patients who met the following criteria were included: (1) age ≥ 18 years old; (2) patients had cancer confirmed by pathological biopsy or two imaging techniques; (3) seropositive for HBsAg, regularly received antiviral agents and intravenous used at least one cycle of PD-1 inhibitor. According to APASL clinical practice guidelines on hepatitis B reactivation (25), taking NAs for at least one week before receiving PD-1 inhibitors was considered prior use of antiviral agents in this study. Patients were excluded if any of the following occurred during treatment: (1) HAV/HCV/HEV infection; (2) antibodies positive to human immunodeficiency virus (HIV); (3) lack of data on HBsAg quantification before and/or after administration of PD-1 inhibitors.

### Data collection

Demographic data including age and sex were collected. Additional clinical information regarding liver cirrhosis, HBeAg status, serum HBsAg and HBV-DNA levels at baseline (before PD-1 inhibitor initiation) and after PD-1 inhibitor administration, cycles of PD-1 inhibitor, PD-1 inhibitor type (nivolumab, pembrolizumab, sintilimab, toripalimab, tislelizumab, and camrelizumab). The occurrence of irAEs before significant HBsAg changes or HBVr was recorded according to Version 5 of the Common Terminology Criteria for Adverse Events (CTCAE) (26). Prior use of antiviral therapy, antiviral agents (entecavir, tenofovir, tenofovir alafenamide fumarate), combined antineoplastic therapies including chemotherapy, hepatic artery intubation chemotherapy (HAIC), transcatheter arterial chemoembolization (TACE), targeted agents (apatinib, lenvatinib, regorafenib, anlotinib, sorafenib, donafenib), and radiotherapy were obtained. Oncologic factors recorded including cancer type, and ECOG (Eastern Cooperative Oncology Group) score.

### Study design

After the PD-1 inhibitor therapy, eligible patients were divided into two groups based on changes in serum HBsAg levels: the HBsAg decreased group and the HBsAg increased group. The first endpoint was a significant change in serum HBsAg levels, defined as an increase or decrease of more than 0.5 log10-fold in serum HBsAg levels after PD-1 inhibition. Hence, quantification of serum HBsAg needed to be performed at least twice in this study. Most of the serum HBsAg were measured by chemoluminescence technique in the clinical laboratory of our center using an automatic chemiluminescence immunoanalyzer (I 3000; Maccura, SiChuan, China) with a detection range of 0-250 IU/ml. For patients whose serum HBsAg levels were more than 250 IU/ml, the concentrations of serum HBsAg were determined by an electrochemiluminescence immunoanalyzer (COBAS E601; Roche Diagnostics, Basel, Switzerland) with a lower limit of 10-20 IU/ml.

The secondary endpoint was the incidence of HBV reactivation (HBVr). According to the AASLD 2018 hepatitis B guidance, the occurrence of HBVr was defined as (27): for HBsAg- positive patients (1) a 2-log (100-fold) increase in HBV-DNA compared with the baseline levels; (2) HBV-DNA ≥ 3 log (1000-fold) IU/ml in a patient with previously undetectable levels (given that HBV-DNA levels fluctuate); or (3) HBV-DNA ≥ 4 log (10,000-fold) IU/ml if the baseline level was not available. For HBsAg-negative, anti-HBc-positive patients: reverse HBsAg seroconversion occurs (reappearance of HBsAg). HBV-DNA was quantified by real-time polymerase chain reaction (PCR) diagnostic kit (COBAS AmpliPrep/TaqMan; Roche Diagnostics, Basel, Switzerland) with a lower limit threshold of 10 or 20 IU/ml or real-time fluorescence quantitative PCR with a lower limit threshold of 100 IU/ml.

### Statistical analysis

Normally distributed quantitative data were expressed as mean ± standard deviation, and non-normally distributed quantitative data were reported as median (range or interquartile range). Continuous variables were compared using a two-tailed Student’s t-test or Mann-Whitney U test depending on the distribution. The Pearson χ^2^ or Fisher exact test was performed to investigate the relationships between categorical variables. The correlation between pretreatment factors and significant HBsAg decrease or increase and HBVr were evaluated by logistic regression analysis. Factors in the univariable analysis with P < 0.2 were included in the multivariable analysis, a two-tailed P ≤ 0.05 was considered significant. Statistical analysis was performed with SPSS software version 23.0 (SPSS Inc., Chicago, IL, USA).

## Results

### Patient’s characteristics

**Figure 1:**
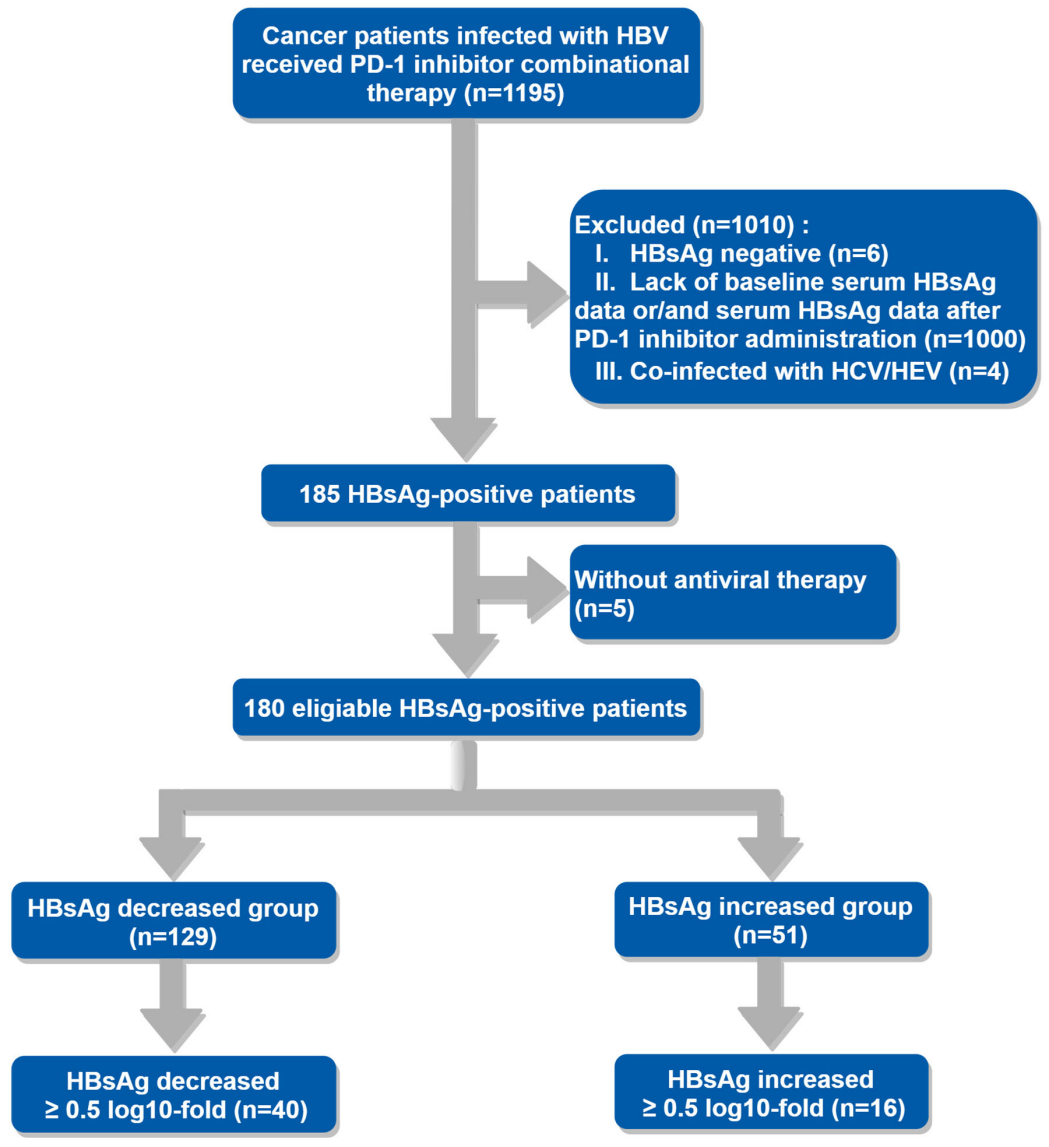
Flowchart showing the process of selecting patients.

185 patients met the inclusion criteria without considering whether they received antiviral therapy or not, only 5 patients didn’t receive antiviral agents during PD-1 inhibitor combinational therapy for unknown reasons. Ultimately, 180 patients who received antiviral treatment were included in the final analysis, the enrollment process was shown in the flowchart (**Figure 1**). The baseline demographic and clinical characteristics of eligible patients are described in **Table 1**. As it presented, more patients in the HBsAg increased group were HBeAg-seropositive (21.43% VS 5.74%, P=0.02). Furthermore, there were differences in antiviral regimens between the HBsAg decreased group and the HBsAg increased group (P=0.03).

**Table 1:** Baseline demographic and clinical characteristics of patients under PD-1 inhibitor combinational therapy.

	HBsAg decreased group (n=129)	HBsAg increased group (n=51)	*P* Value
Age (≤55/>55)	66/63	26/25	0.98
Sex (male/female)	120/9	46/5	0.54
Cancer type	Liver cancer (n=119)	Liver cancer (n=46)	
	Gastric cancer (n=5)	Esophagus cancer (n=2)	
	Biliary duct cancer (n=2)	Lung cancer (n=1)	
	Lung cancer (n=2)	Thymus cancer (n=1)	
	Follicular Lymphoma (n=1)	Urothelia cancer (n=1)	
ECOG score (0/≥1)	63/66	21/30	0.35
Liver cirrhosis (yes/no)	101/28	38/13	0.59
Baseline HBsAg level (IU/ml) (≤500/>500)	111/18	44/7	0.97
Baseline HBV-DNA level (IU/ml) (≤500/>500)	88/41	37/14	0.57
HBeAg status (seronegative, seropositive)	122/7	42/9	0.02
Cycles of PD-1 inhibitor (median, range)	4 (1-23)	5 (1-16)	0.28
PD-1 inhibitor type			0.24
Camrelizumab	27	18	
Sintilimab	69	26	
Toripalimab	3	1	
Tislelizumab	13	2	
PD-1 inhibitor switched*	17	4	
Prior use of antiviral therapy (yes/no)	100/29	40/11	0.89
Antiviral regimen			0.03
ETV	71	27	
TDF	23	18	
TAF	24	5	
NAs switched*	11	1	
Combined with targeted agent			0.76
Sorafenib	7	1	
Lenvatinib	41	16	
Anlotinib	6	5	
Apatinib	9	6	
Regorafenib	8	3	
Donafenib	18	4	
Targeted agent switched*	19	9	
Combined with chemotherapy (yes/no)	13/116	5/46	0.96
Combined with TACE/HAIC (yes/no)	75/54	31/20	0.75
Combined with radiotherapy (yes/no)	12/117	3/48	0.56

ECOG, Eastern Cooperative Oncology Group; HBeAg, hepatitis B envelop antigen; HBsAg, hepatitis B surface antigen; PD-1, programmed cell death protein-1; ETV, Entecavir; TDF, Tenofovir disoprox fumarate; TAF, Tenofovir alafenamide fumarate; NAs, Nucleos(t)ide analogues; HAIC, hepatic arterial infusion chemotherapy; TACE, transcatheter arterial chemoembolization.

Baseline HBsAg (*≤500/>500 IU/ml*) and HBV-DNA level (*≤500/>500 IU/ml*) were referenced from previous studies (12, 17).

PD-1 inhibitor/NAs/targeted agent switched*, the patient switched the type of PD-1 inhibitor/NAs/targeted agent during the observation period.

Patients were predominantly male (n=166, 92.22%), diagnosed with liver cancer (n=165, 91.67%), HBeAg seronegative (n=164, 91.11%), had the background of liver cirrhosis (n=139, 77.22%), and with the mean age of 54.81 ± 10.81 years old. Besides, 15 patients with other types of cancer also were included. Most of the enrolled patients (n=140, 77.78%) started antiviral therapy before PD-1 inhibitor initiation and entecavir (ETV) was selected by over half of the patients (n=98, 54.44%). Among all the patients, only 5 patients (2.78%) received PD-1 inhibitor monotherapy, while most (n=175, 97.22%) adopted PD-1 inhibitor combinational therapy, for instance, combined with chemotherapy (n=18, 10.00%), targeted agents (n=152, 84.44%), TACE or HAIC (n=106, 58.89%) and radiotherapy (n=15, 8.33%), to improve the survival rate of patients. Sintilimab (n=95, 52.78%) was a commonly used PD-1 inhibitor by cancer patients in this study.

### Changes in HBsAg levels after the administration of PD-1 inhibitor under different clinical conditions

**Figure 2:**
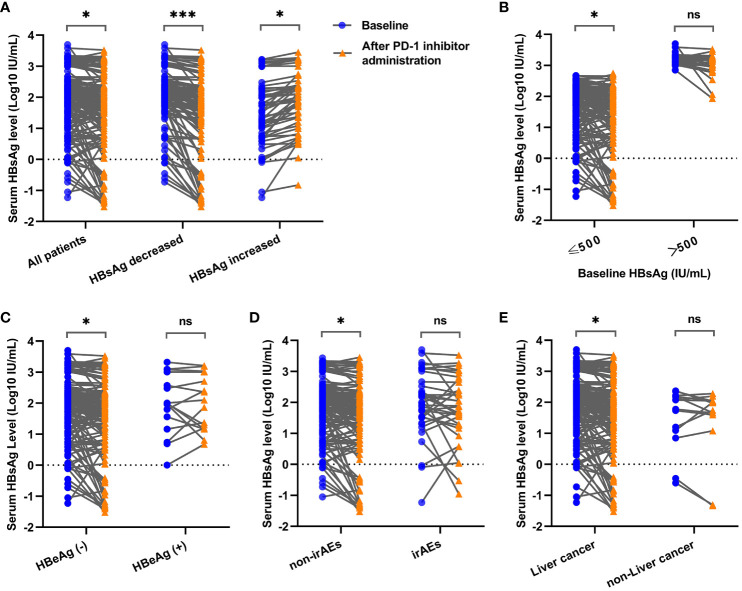
Comparison of serum HBsAg levels before and after PD-1 inhibitor administration in cancer patients under different clinical conditions. **(A)** Comparison of serum HBsAg levels among all enrolled patients, HBsAg decreased group and HBsAg increased group. **(B)** Comparison of serum HBsAg levels in patients with baseline HBsAg ≤ 500 IU/ml and baseline HBsAg > 500 IU/ml. **(C)** Comparison of serum HBsAg levels in HBeAg-seronegative group and HBeAg-seropositive group. **(D)** Comparison of serum HBsAg levels in non-irAEs occurrence group and irAEs occurrence group. **(E)** Comparison of serum HBsAg levels in liver cancer group and non-liver cancer group. * P < 0.05; *** P < 0.001; ns, not statistically significant.

After reviewing the quantitative HBsAg data of patients before and after the initiation of PD-1 inhibitors, an overall decrease in serum HBsAg levels (log10 IU/ml) was observed [2.07 (0.87) VS 1.88 (1.07)] among all enrolled patients (Z=-2.067, P=0.039). Specifically, 129 patients exhibited a decrease [2.22 (0.62) VS 1.85 (1.01)] in serum HBsAg levels (Z=-3.966, P < 0.0001), while 51 patients showed an increase (1.44 ± 1.05 VS 1.84 ± 0.92) in serum HBsAg levels (t=-2.047, P=0.043) under the treatment of PD-1 inhibitors and NAs, as shown in **Figure 2A**. Notably, 40 patients within the HBsAg decreased group and 16 patients within the HBsAg increased group experienced a change in HBsAg levels exceeding 0.5 log10-fold following administration of PD-1 inhibitors.

To investigate the changes of HBsAg under different clinical conditions, multiple subgroups were conducted in the present study. It showed that significant HBsAg decreases were observed in lower baseline HBsAg group (Z=-2.277, P=0.023) (**Figure 2B**), HBeAg-seronegative group (Z=-2.200, P=0.028) (**Figure 2C**), non-irAEs occurrence group (Z=-2.007, P=0.045) (**Figure 2D**) and liver cancer group (Z=-1.987, P=0.047) (**Figure 2E**), while no difference of HBsAg changes was found when patients were divided into groups according to the types of NAs, baseline HBV-DNA levels, liver cirrhosis, prior use of antiviral therapy, the cycles of PD-1 inhibitors, and the types of PD-1 inhibitors (**Supplementary Figure 1**).

### The incidence of serum HBsAg loss in cancer patients

HBsAg loss, defined as a change from positive at baseline to negative at any postbaseline visit within the targeted time window, occurred in 7 patients (7/180, 3.89%), as shown in **Table 2**. All of these patients were male, HBeAg seronegative, and had low baseline HBsAg levels (0.19 to 57.20 IU/ml). 6 patients were diagnosed with liver cancer and liver cirrhosis, and all received antiviral treatment before PD-1 inhibitor. Except for patient 2, who had gastric cancer with no background of liver cirrhosis and without the prior use of antiviral agents. It took 9.29 to 42.86 weeks to achieve HBsAg loss in these patients, only patient 3 experienced HBsAg seroconversion, during which anti-HBs reached 26.30 IU/ml.

**Table 2:** Clinical characteristics of patients with serum HBsAg loss during PD-1 inhibitor combinational therapy.

Patient	Patient 1	Patient 2	Patient 3	Patient 4	Patient 5	Patient 6	Patient 7
Age	61	48	57	70	62	50	52
Gender (male, female)	male	male	male	male	male	male	male
Cancer type	Liver cancer	Gastric cancer	Liver cancer	Liver cancer	Gastric cancer	Liver cancer	Liver cancer
Liver cirrhosis (yes/no)	Yes	No	Yes	Yes	Yes	Yes	Yes
HBeAg status (seronegative/seropositive)	Seronegative	Seronegative	Seronegative	Seronegative	Seronegative	Seronegative	Seronegative
Baseline HBsAg (IU/ml)	0.19	0.25	57.20	1.14	0.35	0.77	1.97
Baseline HBV-DNA (IU/ml)	Not detected	<100	<20	<100	<100	<20	<100
Prior use of antiviral therapy (yes/no)	Yes	No	Yes	Yes	Yes	Yes	Yes
Cycles of PD-1 inhibitor	9	5	11	14	4	2	2
Weeks to achieve HBsAg loss (since PD-1 inhibitor initiation)	26.00	18.86	27.71	42.86	11.43	12.14	9.29
PD-1 inhibitor type	Tislelizumab	Sintilimab	Camrelizumab	Sintilimab	Sintilimab	Tislelizumab Sintilimab	Sintilimab
Antiviral treatment regimen	ETV	ETV	TDF	TAF	TDF	ETV	TDF, TAF
Combined therapy	Lenvatinib	Chemotherapy	Apatinib Oncolytic virotherapy	Donafenib	Chemotherapy	Donafenib TACE	Chemotherapy Lenvatinib
HBV reactivation (yes/no)	No	No	No	No	No	No	No
HBsAg seroconversion (yes/no)	No	No	Yes	No	No	No	No

HBeAg, hepatitis B e antigen; HBsAg, hepatitis B surface antigen; PD-1, programmed cell death protein-1; TACE, transcatheter arterial chemoembolization; ETV, Entecavir; TDF, Tenofovir disoprox fumarate; HBsAg seroconversion, defined as anti-HBs changing from negative at baseline to positive at any postbaseline visit with HBsAg loss occurring within the targeted time window.

### The incidence of HBV reactivation under PD-1 inhibitor combinational therapy

With concurrent use of NAs, HBV-DNA levels were kept undetectable, remained stable at a low level, or decreased in most of the enrolled cancer patients (167/180, 92.78%) in this study. However, there were 11 patients (11/180, 6.11%) developed HBVr within 4.57 to 81.29 weeks under PD-1 inhibitor therapy. The details of these HBV-reactivated patients are listed in **Supplementary Table 1**. HBV-DNA levels of 9 patients increased by at least 100-fold compared to baseline, and the highest HBV-DNA level was 2.54×10^8^ IU/ml at the diagnosis of HBVr. Of note, two patients achieved serum HBsAg loss after receiving antiviral agents and PD-1 inhibitors, however, serum HBsAg returned to positive afterward when the PD-1 inhibitor was still being used.

Of all the 11 patients, 7 cases experienced HBVr during PD-1 inhibitor therapy, while HBVr occurred 4.14 to 16 weeks after the last dose of PD-1 inhibitors in the other 4 cases. 3 out of 11 cases were diagnosed with HBV-associated hepatitis, and 2 of them discontinued PD-1 inhibitors due to hepatitis flare and HBV-related acute-on-chronic liver failure (ACLF), respectively. Moreover, we noticed that 5 cases experienced irAEs before HBVr, and 2 of them discontinued PD-1 inhibitors as a result of immunotherapy intolerance. In addition, some patients had withdrawn immunotherapy owing to cancer progression (n=1) and personal willingness (n=3). With the concurrent use of NAs, HBV-DNA levels of 3 cases achieved undetectable, and 7 cases remained detectable in the latest viral quantification, the patient’s condition with HBV-related ACLF worsened and gave up treatment eventually.

### The occurrence of immune-related adverse events, and safety evaluation of PD-1 inhibitors

As confirmed by two physicians, there were 36 (20.00%) patients who had experienced at least one irAEs of any grade during PD-1 inhibitor combinational therapy, and 13 patients (7.22%) developed grade 3/4 adverse events. As shown in **Supplementary Table 2**, the most common adverse event in the present study was rash (n=12, 6.67%), and then followed by hepatitis (n=9, 5.00%), fever (n=4, 2.22%) and hypothyroidism (n=4, 2.22%). The most common grade 3/4 adverse event was hepatitis (n=9, 5.00%). 20 patients received glucocorticoids after the occurrence of irAEs according to clinical guidelines. However, 3 patients didn’t improve due to acute liver failure (ALF), ACLF and acute myocarditis, respectively. During treatment, 11 patients discontinued PD-1 inhibitors permanently due to irAEs, one patient discontinued PD-1 inhibitors due to irAEs and cancer progression. In addition, irAEs didn’t disturb the administration of PD-1 inhibitors in 12 patients but delayed in the rest 12 patients.

To investigate whether the safety of PD-1 inhibitor combinational therapy was related to the baseline HBV-DNA and HBsAg levels, we regrouped patients with reference to previous studies (12, 17), and found that patients with baseline HBV-DNA > 500 IU/ml had a higher percentage of discontinuation of PD-1 inhibitors due to irAEs (OR 1.688 [95% CI, 0.460-6.195], P=0.048). However, there was no difference in the incidence of all-grade irAEs, 3/4 irAEs, HBVr, and HBV-related hepatitis between high and low groups based on baseline HBV-DNA or HBsAg levels as shown in **Table 3**.

**Table 3:** Safety comparison of PD-1 Inhibitor combinational therapy under different grouping conditions.

	Grouped by baseline HBV-DNA (≤500 or >500 IU/ml)				Grouped by baseline HBsAg (≤500 or >500 IU/ml)			
	Low group (n=125)	High group (n=55)	OR (95% CI)	*P* Value	Low group (n=155)	High group (n=25)	OR (95% CI)	*P* Value
irAEs								
All grades	22 (17.60%)	14 (25.45%)	0.626 (0.292-1.340)	0.23	28 (18.06%)	8 (32.00%)	0.469 (0.184-1.193)	0.11
Grade 3/4	9 (7.20%)	4 (7.27%)	0.989 (0.291-3.361)	1.00	10 (6.45%)	3 (12.00%)	0.506 (0.129-1.982)	0.40
HBV reactivation	9 (7.20%)	2 (3.63%)	2.056 (0.429-9.846)	0.51	9 (5.81%)	2 (8.00%)	0.709 (0.144-3.490)	0.65
HBV-associated hepatitis	2 (1.60%)	1 (1.81%)	0.878 (0.078-9.891)	1.00	2 (1.29%)	1 (4.00%)	0.314 (0.027-3.595)	0.36
PD-1 inhibitor disruption due to irAEs								
Discontinuation	5 (4.00%)	7 (12.73%)	0.286 (0.086-0.944)	0.048	9 (5.81%)	3 (12.00%)	0.452 (0.114-1.799)	0.22
Delay	10 (8.00%)	2 (3.64%)	2.304 (0.488-10.886)	0.35	9 (5.81%)	3 (12.00%)	0.452 (0.114-1.799)	0.22

HBsAg, hepatitis B surface antigen; HBV, hepatitis B virus; OR, odds ratio; irAEs, immune-related adverse events; PD-1, programmed cell death protein-1.

### Risk factors associated with significant serum HBsAg fluctuation and HBV reactivation

Considering that there may be minor detection errors or fluctuations in serum HBsAg quantification, we established criteria for defining clinically significant fluctuations in HBsAg levels by referring to a previous study (12). The results of the risk factor analysis are presented in **Tables 4**–**6**.

**Table 4:** Analysis of risk factors associated with significant serum HBsAg decrease during PD-1 inhibitor combinational therapy.

Characteristics	Univariate analysis			Multivariate analysis		
	OR	95% CI	*p* value	OR	95% CI	*p* value
Age ≤ 55	0.641	0.315-1.302	0.22			
Male Gender	3.844	0.485-30.464	0.07			
Liver cancer (*VS* other)	1.156	0.310-4.315	1.00			
ECOG score <1	1.752	0.861-3.565	0.12			
Liver cirrhosis	1.889	0.732-4.877	0.18			
HBeAg-seropositive	0.792	0.214-2.929	1.00			
Baseline HBsAg level ≤500 (IU/ml)	2.299	0.651-8.119	0.19			
Baseline HBV-DNA level ≤500 (IU/ml)	0.860	0.404-1.830	0.70			
Prior use of antiviral therapy	0.980	0.422-2.275	0.96			
Cycles of PD-1 inhibitor >5	1.531	0.754-3.111	0.24			
Combined lines of therapy* <2	0.639	0.308-1.327	0.23			
Occurrence of irAEs	1.215	0.518-2.850	0.65			
Exposure to steroids	1.085	0.333-3.533	1.00			

Eastern Cooperative Oncology Group; HBeAg, hepatitis B envelop antigen; HBsAg, hepatitis B surface antigen; PD-1, programmed cell death protein-1; irAEs, immune-related adverse events.

Combined lines of therapy*, PD-1 inhibitors combined with any one or more than one type of antineoplastic therapy.

The P value of univariate analysis was calculated through Chi-square test or Fisher exact tests; Multivariate analysis was performed through the binary logistic regression.

**Table 5:** Analysis of risk factors associated with significant serum HBsAg increase during PD-1 inhibitor combinational therapy.

Characteristics	Univariate analysis			Multivariate analysis		
	OR	95% CI	*p* value	OR	95% CI	*p* value
Age ≤ 55	0.723	0.257-2.033	0.54			
Male Gender	0.455	0.091-2.283	0.29			
Liver cancer (*VS* other)	0.603	0.123-2.944	0.63			
ECOG score <1	0.489	0.163-1.470	0.20			
Liver cirrhosis	0.874	0.266-2.871	0.76			
HBeAg-seropositive	4.222	1.180-15.112	0.04	7.236	1.757-29.793	0.01
Baseline HBsAg level ≤500 (IU/ml)	3.058	0.389-24.091	0.13			
Baseline HBV-DNA level ≤500 (IU/ml)	0.690	0.237-2.004	0.57			
Prior use of antiviral therapy	0.844	0.257-2.775	0.76			
Cycles of PD-1 inhibitor >5	1.215	0.431-3.426	0.71			
Combined lines of therapy* <2	0.767	0.266-2.209	0.62			
Occurrence of irAEs	2.680	0.904-7.945	0.10	4.077	1.252-13.273	0.02
Exposure to steroids	3.872	1.092-13.725	0.049			

Eastern Cooperative Oncology Group; HBeAg, hepatitis B envelop antigen; HBsAg, hepatitis B surface antigen; PD-1, programmed cell death protein-1; irAEs, immune-related adverse events.

Combined lines of therapy*, PD-1 inhibitors combined with any one or more than one type of antineoplastic therapy.

The P value of univariate analysis was calculated through Chi-square test or Fisher exact tests; Multivariate analysis was performed through the binary logistic regression.

**Table 6:** Analysis of risk factors associated with HBV reactivation during PD-1 inhibitor combinational therapy.

Characteristics	Univariate analysis			Multivariate analysis		
	OR	95% CI	*p* value	OR	95% CI	*p* value
Age ≤ 55	0.337	0.086-1.314	0.10			
Male Gender	0.283	0.054-1.489	0.16			
Liver cancer (*VS* other)	0.903	0.108-7.579	1.00			
ECOG score <1	1.400	0.411-4.765	0.59			
Liver cirrhosis	3.101	0.385-24.970	0.46			
HBeAg-seropositive	1.027	0.123-8.578	1.00			
Baseline HBsAg level ≤500 (IU/ml)	0.709	0.144-3.490	0.65			
Baseline HBV-DNA level ≤500 (IU/ml)	2.038	0.426-9.761	0.51			
Prior use of antiviral therapy	3.000	0.372-24.171	0.46			
Cycles of PD-1 inhibitor >5	1.873	0.549-6.384	0.35			
Combined lines of therapy* <2	0.716	0.202-2.539	0.76			
Occurrence of irAEs	3.710	1.064-12.937	0.045	5.560	1.592-19.420	0.01
Exposure to steroids	2.281	0.451-11.543	0.28			

Eastern Cooperative Oncology Group; HBeAg, hepatitis B envelop antigen; HBsAg, hepatitis B surface antigen; PD-1, programmed cell death protein-1; irAEs, immune-related adverse events.

Combined lines of therapy*, PD-1 inhibitors combined with any one or more than one type of antineoplastic therapy.

The P value of univariate analysis was calculated through Chi-square test or Fisher exact tests; Multivariate analysis was performed through the binary logistic regression.

In the univariable analysis, HBeAg-seropositive (OR, 4.222 [95% CI, 1.180-15.112], P=0.04), and exposure to steroids during treatment (OR, 3.872 [95% CI, 1.092-13.725]; P=0.049) were significant risk factors for HBsAg increase, the occurrence of irAEs (OR, 3.710 [95% CI, 1.064-12.937], P=0.045) was a significant risk factor for HBVr. In the multivariable analysis, HBeAg-seropositive (OR, 7.236 [95% CI, 1.757-29.793], P=0.01) and the occurrence of irAEs (OR, 4.077 [95% CI, 1.252-13.273]; P=0.02) were identified as the independent risk factor for HBsAg increase, the occurrence of irAEs (OR, 5.560 [95% CI, 1.252-13.273], P=0.01) was identified as the only independent risk factor for HBVr. Of note, no significant risk factors were discovered to be associated with significant HBsAg decrease both in univariable and multivariable analysis.

## Discussion

It’s well known that a HBV-DNA decline directly reflects a reduction of viral replication, while HBsAg decline signifies a reduction of transcriptional activity of intranuclear cccDNA and integrated DNA sequences (28). The clearance of HBsAg is regarded as the closest correlate of cure and the ultimate goal of CHB therapy (29). However, only a few clinical trials (11, 12) have attempted to clarify the potential of PD-1/PD-L1 inhibitors in the treatment of CHB. Retrospectively observing changes in HBsAg and HBV-DNA levels in HBsAg-positive cancer patients undergoing PD-1 inhibitor combination therapy may yield more relevant clinical information.

In the present study, we noticed that viral replication could be effectively inhibited in 92.78% (167/180) of enrolled patients, and overall serum HBsAg levels decreased under PD-1 inhibitor and antiviral therapy (P=0.04), which was consistent with the study of Zeng et al. (30), it revealed that HBV targeting gRNA/cas9 induced a decrease in the expression of HBsAg *in vitro*, combined anti-HBV and anti-PD-1 CRISPR/Cas9 exhibited a stronger antiviral effect than either treatment alone. In another WHV study of woodchucks receiving entecavir, anti-PD-L1 mAb prevented viral rebound following withdrawal of entecavir (31). Taken together, it indicated that PD-1 inhibitor combined with NAs played a certain role in inhibiting viral replication and inducing HBsAg decrease. Upon PD-1 blockade, patients with baseline HBsAg ≤ 500 IU/ml were found to have a statistically significant decrease (P=0.02) in serum HBsAg in this study, which was in line with a previous study (32), it demonstrated that HBV-specific T cell functions were better preserved in CHB patients with lower serum HBsAg levels, and PD-L1 blockade improved HBV-specific CD4^+^ T cell function only in HBs^lo^ patients (serum HBsAg < 500 IU/ml). Meanwhile, we noticed that there were 7 patients (7/180, 3.89%) who achieved HBsAg loss, the rate of which was similar to a previous clinical trial (1/24, 4.17%) on CHB (11). However, a recent retrospective study reported that HBsAg seroclearance occurred in only 2 patients (0.39%) out of 511 HBsAg-positive cancer patients undergoing ICIs (13). The discrepancy among studies may be related to the limited patients included in our study, or cancer patients who failed to monitor serum HBsAg regularly in other studies.

It has been reported that the cumulative HBsAg loss rate of HBeAg-positive patients after 7 years of TDF treatment is higher than HBeAg-negative patients (11.8% VS 0.3%) (33), which makes CHB patients, especially HBeAg-negative patients, have to take medication for life. On the contrary, HBeAg-negative patients were prone to experience a decrease in HBsAg levels (P=0.03) in our study, and patients who achieved HBsAg loss were all HBeAg-negative, which may be attributed to the enhancement of HBV-specific T cell function by PD-1 inhibitors (8–10), In addition, the HBsAg levels decreased in the liver cancer group (P=0.047) when compared with the non-liver cancer group (P=0.36), which may be owing to patients with HBV-related liver cancer pay more attention to the regular follow-up of HBV serologic markers, making it easier to observe changes in serum HBsAg levels. Another undeniable fact was that most of the patients included in this study were HBeAg-negative (164/180, 91.11%) and had liver cancer (165/180, 91.67%), resulting in a more significant statistical difference in these patients.

Consistent with other studies, HBVr (11/180, 6.11%) was also discovered in this study. However, the incidence of HBVr varied greatly (0-30.05%) in different studies (34). The discrepancy may lie in the differences in the proportion of patients who received PD-1 inhibitor monotherapy versus combination therapy. Additionally, unlike the present study, other studies also included HBsAg-negative cancer patients. Even though no correlation was found between HBVr and combined lines of therapies in both univariate and multivariate analysis in this study, PD-1 inhibitor itself, chemotherapy, targeted agent, TACE (35), HAIC (17), and radiotherapy (36) had all been reported to pose a risk of HBVr in cancer patients. Of note, two patients first experienced HBsAg loss, followed by a re-positivity of HBsAg. This suggests that the stability of HBsAg loss induced by PD-1 inhibitors may be unstable or susceptible to other combination therapies. Besides, one patient experienced PD-1 inhibitor discontinuation due to HBV-related ACLF and had a poor prognosis, which reflected that HBVr posed unique challenges to the oncologic population including the possibility of treatment delays or discontinuation of systemic therapies that may affect overall survival. However, with additional awareness, screening, and appropriate antiviral prophylactic, most cases of HBVr can be prevented and well managed (37).

To the best of our knowledge, the present study first identified the occurrence of irAEs as the only independent risk factor for HBVr, while failed to find any factors associated with HBVr that had been reported in other studies including male sex, younger age, HBeAg-seropositive, the presence of cirrhosis (38, 39) and PD-1 inhibitor combined with HAIC (17), etc. The reason for this discrepancy may be attributed to an imbalanced gender distribution in our study, as well as the older age, predominantly HBeAg-seronegative status, and presence of liver cirrhosis among patients with HBVr in the present study. Additionally, a larger proportion of patients received HAIC in the previous study. Furthermore, researchers rarely considered the possible causal relationship between irAEs and HBVr. The possible mechanism of HBVr triggered by PD-1 inhibitor might be that: i) blocking the PD-1/PD-L1 axis may lead to the destruction of hepatocytes and the release of previously latent virus into circulation (40). ii) PD-1 blockade may promote the proliferation of T regulatory cells (Tregs) (41) and myeloid-derived suppressor cells (MDSCs) (42), increasing immuno-suppression and then the reactivation of HBV; iii) MDSC levels were considered as a novel biomarker for related immune dysfunction, such as irAEs (43), and inflammatory Treg reprogramming was suggested a feature of immunotherapy-induced irAEs (44), this may explain that irAEs occurrence was a risk factor for HBVr.

What also can’t be ignored in the present study was that serum HBsAg levels increased (P=0.043) in 51 cancer patients, HBeAg-seropositive and the occurrence of irAEs were identified as the independent risk factors for significant HBsAg increase. The underlying mechanism for this may be: i) T cells, B cells, NK cells, and DCs were associated with the clearance of serum HBsAg (45), impairing these immune cells through cytotoxic drugs, which were used in combinational therapies such as chemotherapy, TACE and HAIC, may lead to the increase of HBsAg; ii) The HBeAg-seropositive patients included in this study were mostly in the immune clearance phase, a typical feature of this phase was the occurrence of spontaneous flares, which were often preceded by an increase in the HBV-DNA level (46), and a positive correlation between pHBsAg (the percentage of immunohistochemical HBsAg) and serum levels of HBV-DNA and HBsAg were observed by another study (47), especially in HBeAg-seropositive group. iii) as the suppression of excessive functions of Tregs and MDSC may be one of the proposed immune mechanisms for HBsAg seroclearance (45), the involvement of these cells in irAEs may lead to an increase in HBsAg levels. However, a negative correlation between the Treg frequency and irAEs was discovered by preclinical models of irAEs (48), and the frequency of peripheral Tregs between irAEs group and non-irAEs group showed no significant differences in patients with advanced metastatic melanoma who were receiving PD-1 inhibitors (44), which implied the controversial role that Tregs played in irAEs. Therefore, more detailed studies should be conducted to explore the immune mechanisms underlying HBVr or HBsAg increase under PD-1 inhibitor therapy, as well as to elucidate the paradoxical role of Tregs in irAEs.

With the increasing use of ICIs, cancer patients are at risk of a series of irAEs that can present at any time, including after cessation of immune checkpoint blockade therapy, and may wax and wane over time (1). In this study, 20% (36/180) of patients experienced all-grade irAEs and 7.22% (13/180) of patients developed severe irAEs (grade 3/4), which resulted in delayed and discontinued use of PD-1 inhibitors. Inconsistent with previous studies (19, 20), our study showed a lower prevalence of irAEs in cancer patients. This may be related to the difficulty of evaluating profiles of irAEs and obtaining accurate data on incidence or prevalence, due to selection criteria, relatively small sample sizes, strict diagnosis standards, and limited duration of follow-ups. In addition, we noticed that HBsAg levels were decreased (P=0.045) in the non-irAEs group compared to the irAEs group, which indirectly supported that the occurrence of irAEs was a risk factor for elevated serum HBsAg levels. Interestingly, we also noticed that patients with baseline HBV-DNA > 500 IU/ml had a higher rate of discontinuation of PD-1 inhibitors (P=0.048) due to irAEs. This may be partially attributed to the higher irAEs incidence in patients with baseline HBV-DNA > 500 IU/ml in this study, meanwhile, the patient’s acceptance and tolerance of irAEs also should be considered. Although studies (11, 21, 22, 49) have shown that PD-1/PD-L1 inhibitors are relatively safe and effective for cancer patients, we should still be cautious of the irAEs they may cause. Given the high immunogenicity and long half-life of PD-1 or PD-L1 therapeutic blocking mAbs, they are more likely to cause higher levels of irAEs and are difficult to be timely removed (50). Recently, Zhai et al. (51) have demonstrated a newly screened cyclic peptide C8, which can be removed in a shorter period of time to reverse the irAEs due to its reasonable half-life, could work as a blocker for PD-1 and reactivate CD8^+^ T cells to treat cancers. It may have the potential as a drug candidate not only for cancer immunotherapy but also for treating chronic hepatitis B in the future.

## Conclusion

Under the concurrent use of NAs, we observed an overall decrease in the levels of serum HBsAg in cancer patients receiving PD-1 inhibitor combinational therapy, with a small number of patients achieving HBsAg loss, and the viral replication of most patients can also be effectively inhibited. It suggested that PD-1 inhibitors combined with NAs may have therapeutic effects on chronic HBV infection, and may contribute to the clinical cure of hepatitis B. However, due to the influence of the PD-1 inhibitor itself or other combined antineoplastic therapies, the state of HBsAg loss in some patients cannot be stably maintained.

Except that HBeAg-positive was identified independent risk factor for significant HBsAg increase, our study first identified the occurrence of irAEs as the independent risk factor both for significant HBsAg increase and HBVr, and patients may discontinue PD-1 inhibitors as a result of HBVr or irAEs. This may provide some risk implications for researchers conducting clinical trials using PD-1 or PD-L1 inhibitors to treat CHB, and clinicians need to pay more attention to the safety of PD-1 inhibitors.

## Limitations

However, there are several limitations in this study. First, most of the cancer patients with HBV infection are excluded for lacking the awareness of monitoring serum HBsAg or HBV-DNA regularly, which may lead to selection bias, more eligible patients should be enrolled in future studies. Second, more well-designed, large-scale prospective and retrospective studies on cancer patients with HBV infection are needed before any definitive conclusions can be reached. Third, there were few patients with other types of cancer included in this study, more patients diagnosed with other types of cancer should be enrolled in future studies. Fourth, although the quantitation of serum HBsAg and HBV-DNA levels, particularly serum HBsAg levels, were mostly performed using the same quantitative methods before and after PD-1 inhibitor administration in this study, it is essential for the quantitative methods of serum HBV-DNA and HBsAg to remain consistent throughout the treatment.

## Data availability statement

The original contributions presented in the study are included in the article/**Supplementary Material**. Further inquiries can be directed to the corresponding authors.

## Ethics statement

The studies involving humans were approved by the institutional review board of the Third Affiliated Hospital of Sun Yat-sen University. The studies were conducted in accordance with the local legislation and institutional requirements. The ethics committee/institutional review board waived the requirement of written informed consent for participation from the participants or the participants’ legal guardians/next of kin because of the retrospective nature of this study.

## Author contributions

YZ: Data curation, Investigation, Methodology, Visualization, Writing – original draft. JH: Data curation, Investigation, Resources, Writing – review & editing. JP: Data curation, Investigation, Methodology, Writing – review & editing. SP: Data curation, Investigation, Methodology, Writing – review & editing. YJ: Data curation, Resources, Writing – review & editing. YW: Data curation, Resources, Writing – review & editing. XL: Conceptualization, Formal analysis, Project administration, Supervision, Writing – review & editing. YC: Conceptualization, Formal analysis, Project administration, Supervision, Writing – review & editing.

## Supplementary material

Supplementary Figure 1 Comparison of serum HBsAg levels before and after PD-1 inhibitor administration in cancer patients under different clinical conditions. **(A)** Comparison of serum HBsAg levels in patients received different antiviral agents. **(B-D)** Comparison of serum HBsAg levels in patients with different baseline HBV-DNA levels. **(E)** Comparison of serum HBsAg levels in liver cirrhosis group and non-liver cirrhosis group. **(F)** Comparison of serum HBsAg levels in patients with and without prior use of antiviral therapy. **(G)** Comparison of serum HBsAg levels in patients received PD-1 inhibitors ≤ 5 cycles and > 5 cycles. **(H)** Comparison of serum HBsAg levels in patients under different types of PD-1 inhibitor therapy. ns, not statistically significant; ETV, Entecavir; TDF, Tenofovir disoprox fumarate; TAF, Tenofovir alafenamide fumarate. Due to limited cases, serum HBsAg levels were not compared in patients under Toripalimab therapy and NAs switched therapy.
