# Supplementary figures and images for: The occurrence of immune-related adverse events is an independent risk factor both for serum HBsAg increase and HBV reactivation in HBsAg-positive cancer patients receiving PD-1 inhibitor combinational therapy
Source: Front Immunol. 2024 Mar 15;15:1330644. doi: 10.3389/fimmu.2024.1330644 (PMC10979302; doi:10.3389/fimmu.2024.1330644)

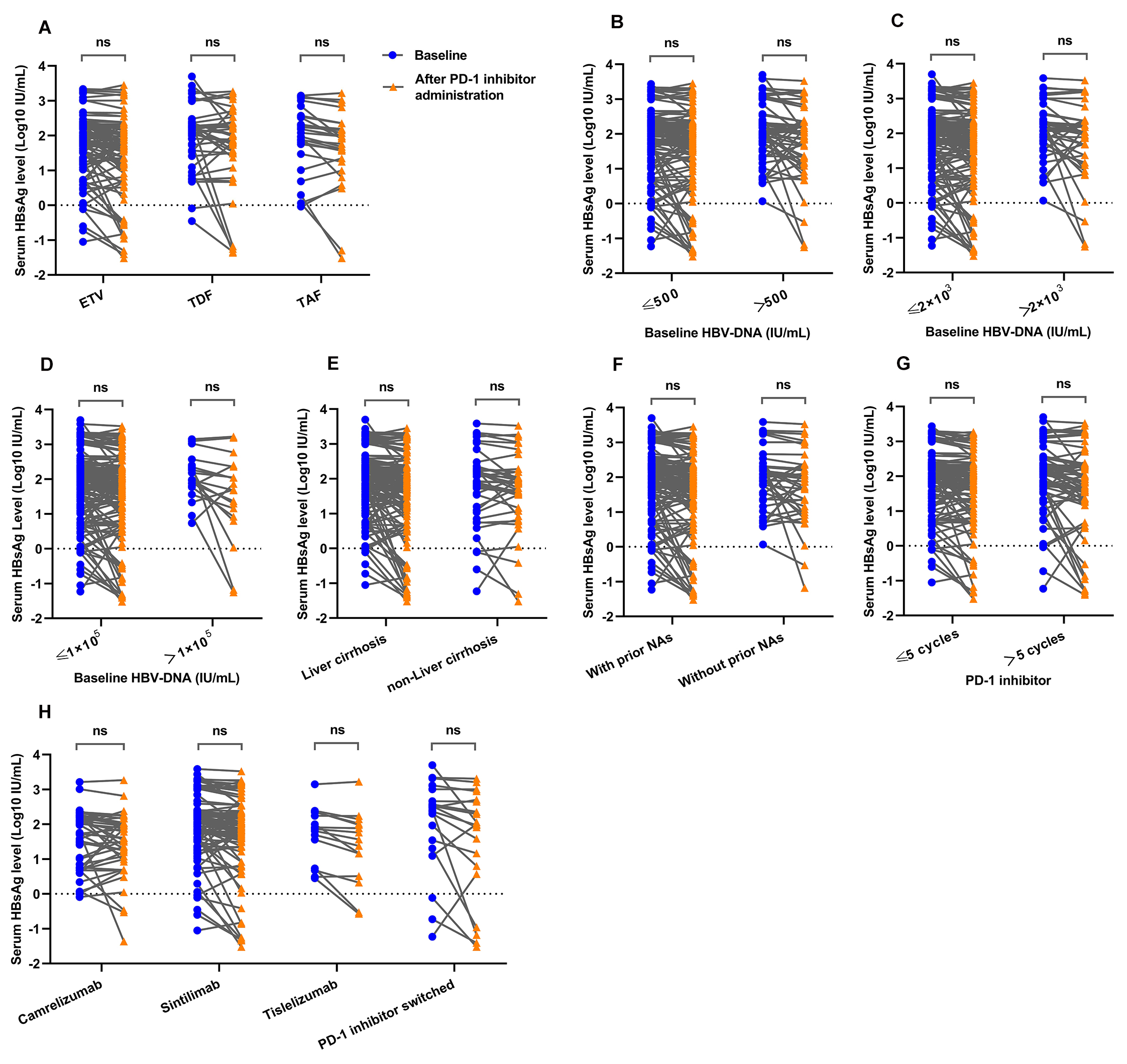

Supplement: Supplementary Figure 1 — Comparison of serum HBsAg levels before and after PD-1 inhibitor administration in cancer patients under different clinical conditions. (A) Comparison of serum HBsAg levels in patients received different antiviral agents. (B-D) Comparison of serum HBsAg levels in patients with different baseline HBV-DNA levels. (E) Comparison of serum HBsAg levels in liver cirrhosis group and non-liver cirrhosis group. (F) Comparison of serum HBsAg levels in patients with and without prior use of antiviral therapy. (G) Comparison of serum HBsAg levels in patients received PD-1 inhibitors ≤ 5 cycles and > 5 cycles. (H) Comparison of serum HBsAg levels in patients under different types of PD-1 inhibitor therapy. ns, not statistically significant; ETV, Entecavir; TDF, Tenofovir disoprox fumarate; TAF, Tenofovir alafenamide fumarate. Due to limited cases, serum HBsAg levels were not compared in patients under Toripalimab therapy and NAs switched therapy. [file Image_1.jpg]
